# Characterisation of the Morphological, Functional and Molecular Changes in Sunitinib-Resistant Renal Cell Carcinoma Cells

**DOI:** 10.15586/jkcvhl.2018.106

**Published:** 2018-08-10

**Authors:** Hossam Kamli, Glenda C. Gobe, Li Li, David A. Vesey, Christudas Morais

**Affiliations:** 1 Translational Research Institute, University of Queensland Princess Alexandra Hospital Kidney Disease Research Collaborative , Brisbane, Queensland, Australia; 2 Department of Clinical Laboratory Sciences, College of Applied Medical Sciences, King Khalid University, Abha, Saudi Arabia; 3 UQ NHMRC CKD.QLD CRE, Royal Brisbane and Women’s Hospital, Brisbane, Australia; 4 UQ-Ochsner Clinical School, Translational Cancer Research Laboratory, Institute of Translational Research, Ochsner Clinic Foundation, New Orleans, LA, USA; 5 Department of Renal Medicine, Princess Alexandra Hospital, Woolloongabba, Brisbane, Australia; 6 Department of Urology, Princess Alexandra Hospital, Woolloongabba, Brisbane, Australia

**Keywords:** angiogenesis, anti-apoptosis, interleukin-6, renal cell carcinoma, sunitinib resistance

## Abstract

Sunitinib resistance is a major clinical problem hampering the treatment of renal cell carcinoma (RCC). Studies on the comprehensive characterisation of morphological, functional and molecular changes in sunitinib-resistant RCC cells are lacking. The aim of the current study was to develop sunitinib resistance in four human RCC cell lines (786-0, Caki-1, Caki-2 and SN12K1), and to characterise the changed cell biology with sunitinib resistance. RCC cells were made resistant by continuous, chronic exposure to 10 μM of sunitinib over a period of 12 months. Cell proliferation, morphology, transmigration, and gene expression for interleukin-6 (IL-6), interleukin-8 (IL-8), vascular endothelial growth factor (VEGF), Bcl-2 and Bax were studied. There was no significant difference in growth rate or transmigration between the parental and resistant cells. Sunitinib-resistant cells were significantly hypertrophic compared with parental cells as evidenced by increases in the surface areas of the whole cells and the nuclei. IL-6 was significantly increased in all resistant cells. IL-8 was increased in sunitinib-resistant Caki-2 and SN12K1 cells and decreased in 786-0 without any significant changes in Caki-1. VEGF was increased in resistant Caki-2 and SN12K1 cells but not in 786-0 and Caki-1. The Bcl2/Bax ratio was increased in Caki-1, Caki-2 and SN12K1 cells but decreased in 786-0 cells. The increased IL-6 may contribute to sunitinib resistance either via VEGF-mediated angiogenesis or through shifting of the Bcl2/Bax balance in favour of anti-apoptosis.

## Introduction

Vascular endothelial growth factor receptor (VEGFR)-targeted tyrosine kinase inhibitors (TKIs) have become the mainstay of treatment for metastatic renal cell carcinoma (RCC). Sunitinib is one of the first-line TKIs (1–5) that targets multiple receptor tyrosine kinases such as VEGFR-1, VEGFR-2 and VEGFR-3; platelet-derived growth factor receptor alpha (PDGFR)-α and (PDGFR)-β; stem cell growth factor receptor (KIT); fms-related tyrosine kinase 3 (FLT3); glial cell line–derived neurotrophic factor receptor (RET); and colony-stimulating factor receptor 1 (CSF1R) (6–9). Sunitinib targets not only endothelial cells and the endothelial proangiogenic factors (9) but also the tumour cells (8), leading to inhibition of angiogenesis and regression of tumours. As with most chemotherapeutics, resistance to sunitinib is a concern. About 30% of patients are thought to be inherently resistant to sunitinib (10–14) and the remaining 70% who initially respond will eventually develop acquired resistance during the course of the treatment, usually within 12 months (10–13, 15–17).

Many in vitro studies have attempted to elucidate the mechanisms of acquired resistance to sunitinib. Based on current knowledge, the mechanisms behind sunitinib resistance can be grouped under two major categories: reduced bioavailability and activation of alternate angiogenesis pathways. Reduced bioavailability is mediated either through the sequestration of sunitinib in lysosomes or through ral-interacting protein 76 (RLIP76) transporters and sphingosine kinase-1 (SK1)-mediated efflux (18–20). Activation of alternate angiogenesis pathway is the result of a myriad of molecules including ATX (autotaxin) (21), chemokines (22), Cox-2 (cycloxygenase-2) (23), EMMPRIN (extracellular matrix metalloproteinase inducer) (24), HDM2 (human double minute 2), HDMX (human double minute x) (25), IL-8 (26), IL-6 (27, 28), LPA (lysophosphatidic acid) (21), MDSC (myeloid-derived suppressor cells) (29), NGAL (neutrophil gelatinase-associated lipocalin) (30), PRKX (protein kinase x-linked) (31), PTEN (phosphatase and tensin homolog) (32), microRNAs (33) and many more emerging molecules and signalling pathways. In addition to the molecular changes, sunitinib may induce morphologic changes to RCC cells, for example, changes indicative of epithelial–mesenchymal transition (34). Despite these, to the best of our knowledge, studies on comprehensive characterisation of the morphological, functional and molecular changes in sunitinib-resistant RCC cells are lacking. In the current study, we established four human RCC cell lines that are resistant to sunitinib, and characterised their morphological, functional and possible molecular mechanisms of sunitinib resistance.

## Materials and Methods

### Cell culture

The RCC cell lines 786-0, Caki-1 and Caki-2 were obtained from American Type Culture Collection (Rockville, MD). Another human RCC cell line, SN12K1, was obtained from Professor D Nicol, Princess Alexandra Hospital, Brisbane, Australia, through his collaborations with Dr IJ Fidler, Cancer Research Institute, MD Anderson Cancer Center, Houston, TX, USA. The RCC cell lines were cultured in DMEM/F12 (Gibco, Invitrogen, CA, USA) supplemented with foetal bovine serum (10%), penicillin (50 U/ml), streptomycin (50 μg/ml) and amphotericin B (0.125 μg/ml) in a humidified atmosphere of 5% CO_2_ in air at 37^°^C. All cell lines were recurrently tested and determined to be mycoplasma-free.

### Development of sunitinib-resistant RCC cell lines

Cells resistant to 10 μM sunitinib were established by exposure to increasing concentrations of sunitinib. In brief, the RCC cell lines were treated with varying concentrations of sunitinib (0, 1, 5, 10, 20, 50 and 100 μM). While all concentrations above 1μM inhibited the growth rate of the RCC cell lines, at 10 μM, more than 98% of cells were dead by 72 h, as measured by MTT assay. It was assumed that the remaining cells were a mix of transient (or tolerant) and stable resistant cells. If this assumption is true, with the passage of time, the transient cells are eliminated, and only stably resistant cells would eventually grow to confluence. With this assumption, these cells were continually maintained in 10 μM sunitinib and passaged every 4 days and the cells that eventually grew to confluence were developed into stable sunitinib-resistant cells over a period of 12 months. At no point during the development process were the cells in sunitinib-free medium. Further experiments showed that these cells were also resistant to 20 and 40 μM sunitinib. However, experiments were conducted in 10 μM. The results presented are from sunitinib-resistant cells that had been in culture for more than 12 months.

### Measurement of cell and nuclear size as a marker of hypertrophy

The surface area of whole cell and nucleus, as a marker of hypertrophy, was analysed as per previous reports (35, 36). In brief, parental and resistant cells were seeded and cultured on glass cover slips in 24-well plates at a density of 4 × 10^4^ cells/ml. After 24 h, the cells were washed in phosphate-buffered saline (PBS), fixed for 20 min at room temperature in 4% formaldehyde, stained with haematoxylin and eosin as per routine methodology and mounted with DePex mounting medium. The cells were viewed under a Nikon Eclipse 50i microscope (Nikon Instruments Inc., NY, USA) at 200× magnification. Images were captured from four random areas of each coverslip using DS-Fi1 colour camera (Nikon Australia, Sydney, Australia). Analyses of cell and nuclear sizes were performed using NIS Elements Software version 2.0 (Nikon Instruments, Melville, NY, USA).

### Cell growth assay using MTT

Cells were seeded in 96-well plates (5 × 10^3^ cells/well/100 μl) and MTT assay was performed as per previous reports (35–37). After pre-determined time periods (24 h–120 h), 5 μl of MTT (Sigma, MO, USA), from a 5 mg/ml stock in PBS, were added to each well of the culture plates and incubated for 90 min at 37^°^C in a humidified atmosphere of 95% air and 5% CO_2_. The culture medium was removed, and the purple crystals formed were dissolved in 100 μl of dimethyl sulfoxide (DMSO). The absorbance was read at 570 nm with a background correction of 690 nm in a Multiscan Go Microplate Reader (Thermo Scientific, Waltham, MA, USA). The percentage of cell viability was calculated relative to the control wells, which were designated as 100%.

### Transmigration

Transmigration assay was performed using transwell migration chambers following the protocol of the supplier (Thermo Fisher Scientific Australia Pty Ltd; Cat # NUN140629; 8 μm pore size). Briefly, the cells were suspended in serum-free DMEM/F12 medium and seeded on the upper compartment of the transwell. Five hundred microlitres of culture medium containing 10% FBS were added as chemoattractant to the bottom chamber. After 24 h, non-migrated cells from the upper surface of the membrane were removed using a wet cotton swab. The migrated cells on the lower surface of the membrane were fixed in 4% formaldehyde, stained with toluidine blue (1% in a 1% aqueous solution of borax) and mounted with DePex mounting medium. The cells were viewed under a 40 × objective and counted from five random fields.

### Gene expression studies

Total RNA was isolated from cells using PureLink RNA Mini Kit as per the manufacturer’s instructions (Life Technologies, Carlsbad, CA, USA). After quantifying with Nano drop (Thermoscientific, Waltham, MA, USA), cDNA was synthesised using a high capacity cDNA Reverse Transcription Kit (Life Technologies). In brief, 1 μg of RNA in reaction mixture (2 μL 10× buffer, 0.8 μL dNTP, 2 μL r-hex, 1 μL enzyme, water to 20 μL per reaction) was subjected to the following PCR conditions: 25^°^C for 10 min, 37^°^C for 60 min, 37^°^C for 60 min, 80^°^C for 5 min followed by holding at 4^°^C. The cDNA was diluted to 50 μL with 30 μL of RNAse-free water. Fully validated TaqMan Gene Expression Assay (Life Technologies) for Bax (Hs00180269_m1), Bcl2 (Hs00236808_m1), IL-6 (Hs00985639_m1), IL-8 (Hs00174103_m1) and VEGFA (Hs00900055_m1) was used with the SensiFAST™ Probe No-ROX Kit (Bioline, London, UK) in a LightCycler 480 (Roche Applied Science, Penzberg, Germany) to determine relative gene expression by the comparative Ct method. TBP (TATA box binding protein; Hs00427620_m1) was used as an internal control and for normalisation (35–37).

### Statistical analyses

The results were expressed as mean ± standard deviation of mean. Comparisons between groups were analysed using analysis of variance (ANOVA) with Tukey’s post hoc test or Student’s *t*-test, where appropriate. Analyses were performed using Graphpad Instat software (San Diego, CA, USA). A *p*-value of <0.05 was considered significant. The results presented are representatives of three independent experiments.

## Results

### Morphological changes

**Figure 1.**
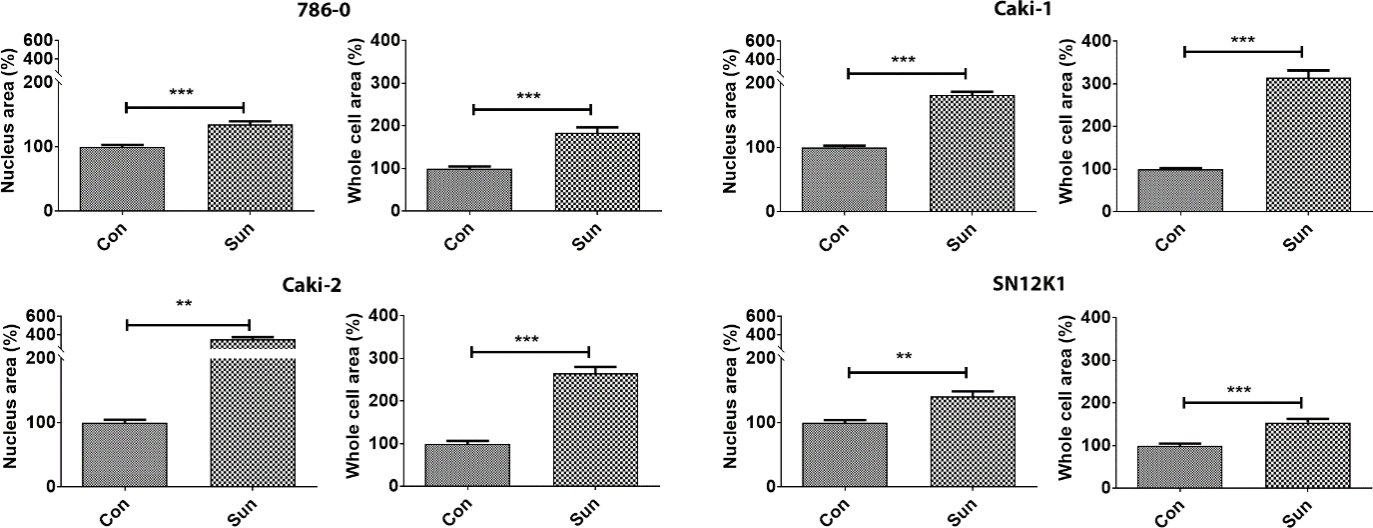
Sunitinib-resistant cells are hypertrophic. RCC cell lines that are resistant to 10 μM of sunitinib showed significant increases in the surface areas of the whole cells and the nuclei. Results are presented as percentage of the control group. **P < 0.01 and ***P < 0.001.

**Figure 2.**
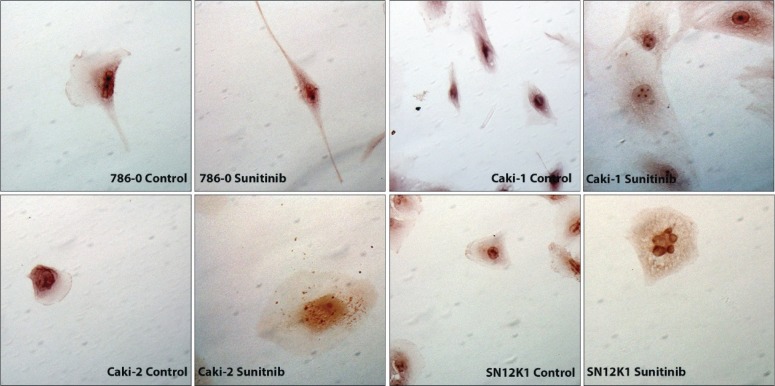
Morphological heterogeneity and nuclear atypia in sunitinib-resistant cells. Representative H&E images showing increased cell and nuclear size in resistant cells. The 786-0 appeared spindle-shaped whereas the SN12K1 showed multi-lobular nucleus or nuclear blebs.

**Figure 3.**
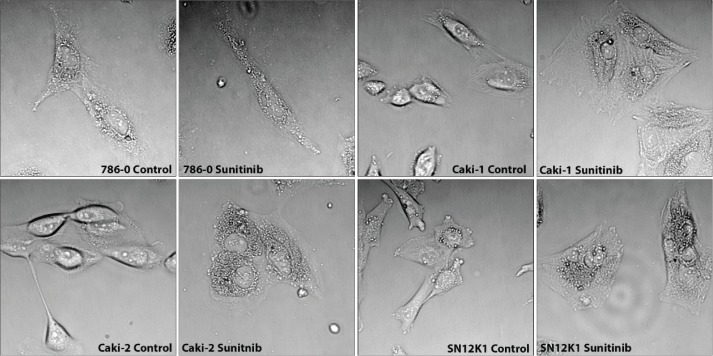
Phase contrast microscopy of live cells showing hypertrophy of sunitinib-resistant cells. Representative phase contrast microscopy further confirms the hypertrophic nature of sunitinib-resistant cells.

Morphometric studies showed significant hypertrophy in sunitinib-resistant cell lines, as evidenced by increases in the surface areas of the whole cells and the nuclei (Figure 1). Representative H&E stained cells from each group are shown in Figure 2. While all cell types were hypertrophic, considerable morphologic heterogeneity could be observed in the resistant cells. In particular, 786-0 showed spindle morphology, whereas the SN12K1 cells displayed multi-lobulated nuclei. Observation of live cells under phase contrast microscope further confirmed hypertrophic and heterogenic features of resistant cells (Figure 3).

### Growth and transmigration

**Figure 4.**
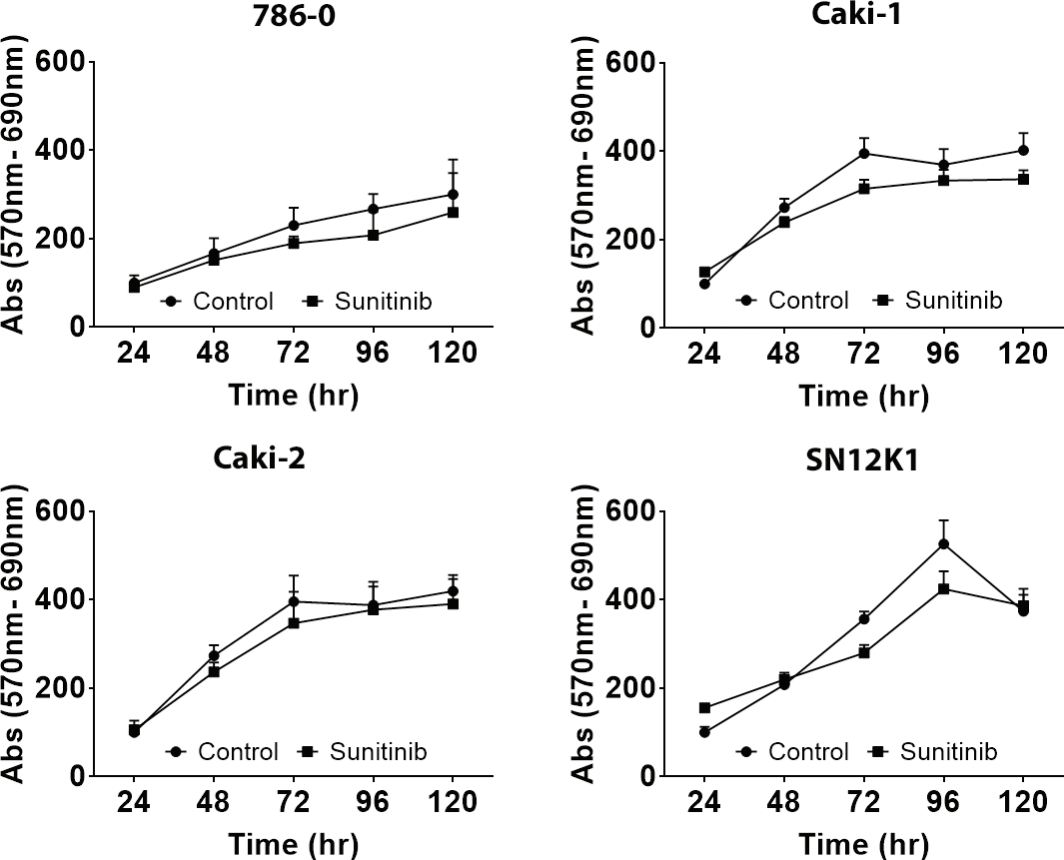
Growth rate showed no significant difference between control and sunitinib-resistant cells. In general, resistant cells showed a slightly decreased growth rate but this was not statistically significant. Results are expressed as percentage of control.

**Figure 5.**
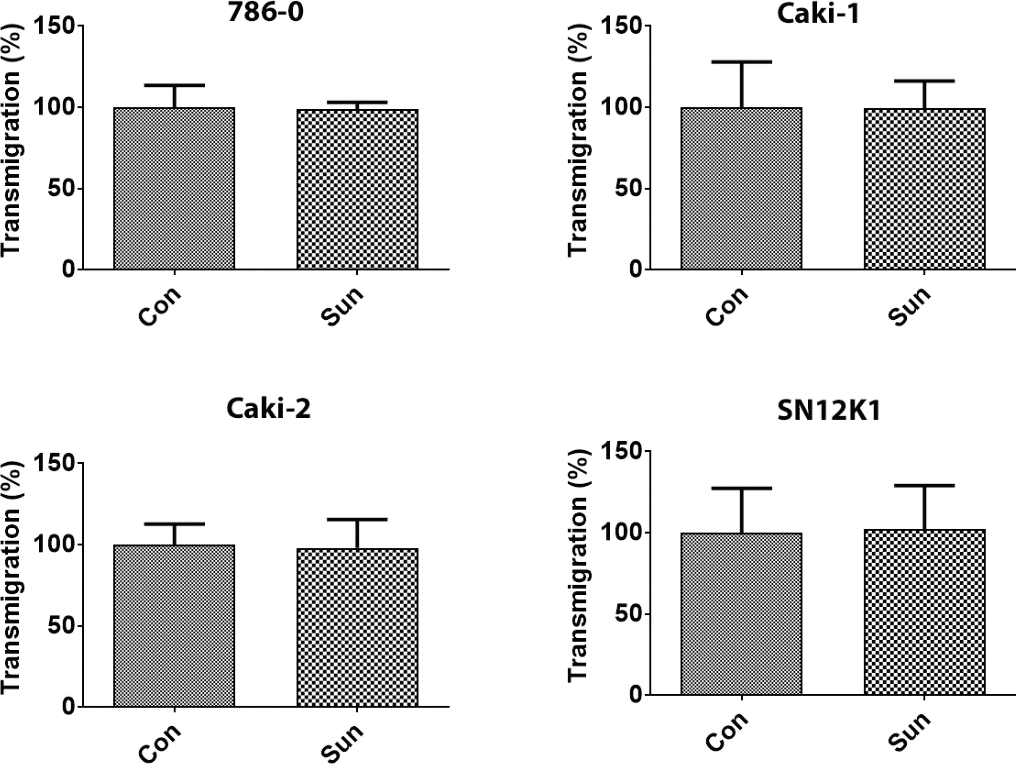
Lack of difference in transmigration between control and sunitinib-resistant cells. After 24 h of transmigration, in response to 10% FBS as a chemoattractant, no statistically significant difference was observed between the parental and sunitinib-resistant cells. Results are expressed as percentage of control.

Although the resistant cells showed a slightly decreased growth rate, at no point in time during the course of the study was this difference statistically significant (Figure 4). Similarly, there was no statistically significant difference in transmigration between the parental and sunitinib-resistant cells (Figure 5). Whether this lack of difference is the reflection of increased size of the sunitinib-resistant cells preventing them from migrating or actual decrease in transmigration is not clear.

### Gene expression

**Figure 6.**
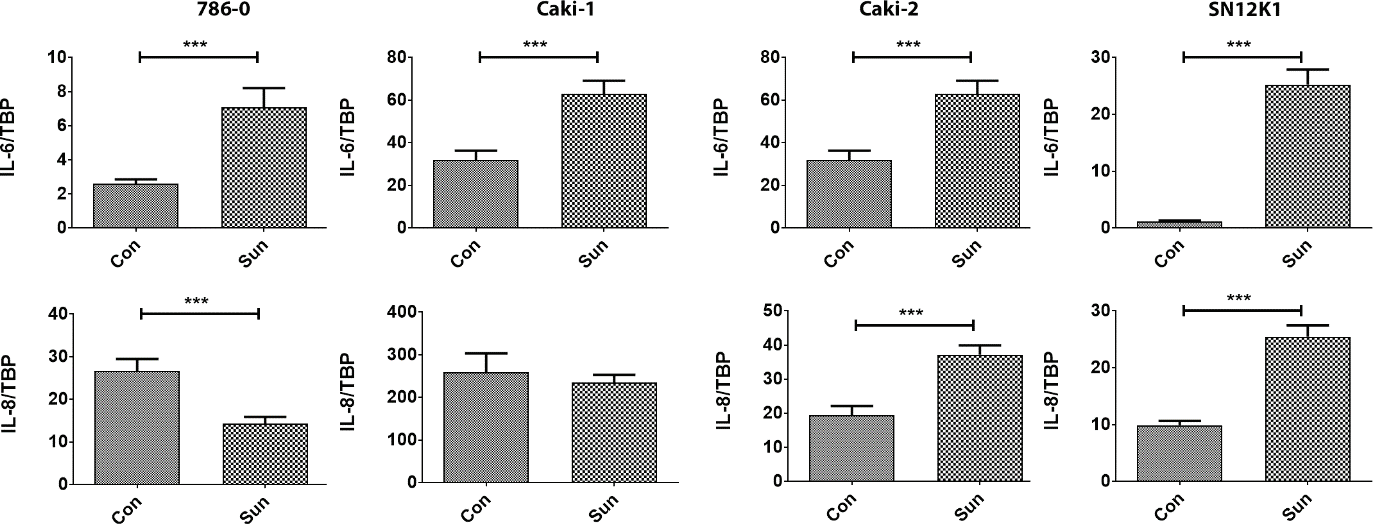
IL-6 is increased in all resistant cells. IL-6 was the common molecule that was increased in all sunitinib-resistant cells. Regarding IL-8, heterogeneity in expression was observed. ***P < 0.001.

**Figure 7.**
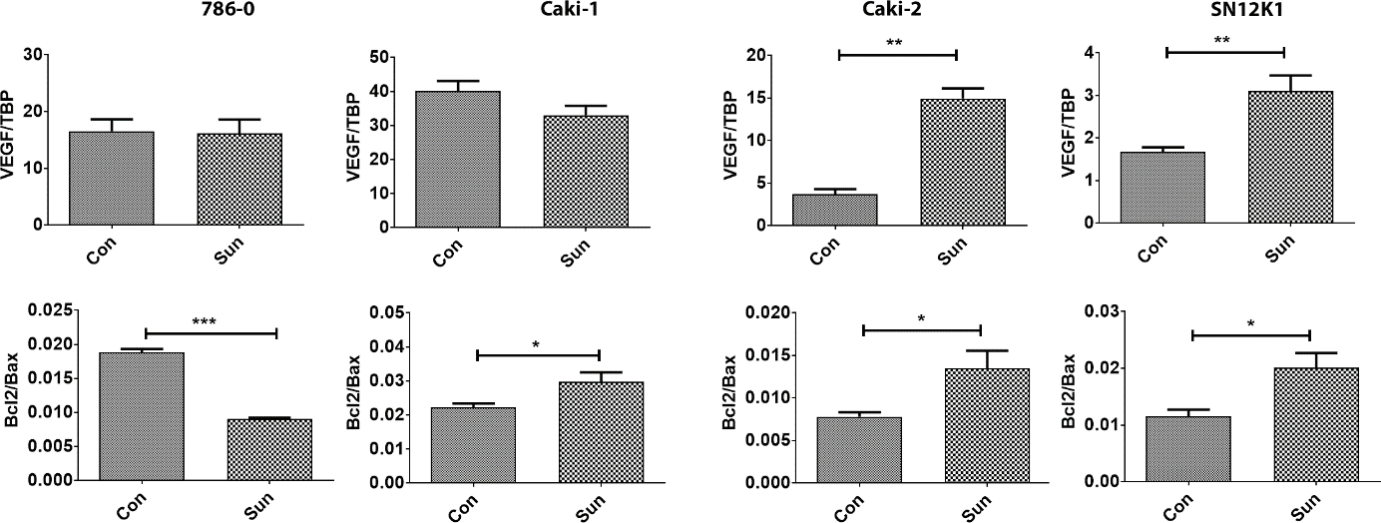
Differential expression of proangiogenic and apoptosis-regulatory genes in resistant cells. There was an increased expression of the proangiogenic gene VEGF in Caki-2 and SN12K1 cells and an increased Bcl2/Bax ratio in Caki-1, Caki-2 and SN12K1 cells. *P < 0.05, **P < 0.01 and ***P < 0.001.

To investigate the possible mechanisms behind resistance, we studied the expression patterns of the proangiogenic factors IL-6, IL-8 and VEGF, and apoptosis-regulatory molecules Bcl-2 and Bax. In all resistant cells, IL-6 was the only common molecule that was overexpressed (Figure 6). IL-8 was increased in Caki-2 and SN12K1 cells and decreased in 786-0. No significant change was observed in Caki-1. Interestingly, VEGF was increased in resistant Caki-2 and SN12K1 cells, without any significant changes in 786-0 and Caki-1 (Figure 7). The Bcl2/Bax ratio was increased in Caki-1, Caki-2 and SN12K1 cells but decreased in 786-0 cells (Figure 7).

## Discussion

There is no universal consensus or guidelines on how drug-resistant cells should be developed in vitro. The underlying principle is to develop cells that are resistant to concentrations that would otherwise kill the parental cells. There are many methods, each with its own advantages and disadvantages, and the choice is often at the discretion of the investigators. Furthermore, there is no agreement on the terminology on whether it is drug tolerance or resistance, which also varies from investigator to investigator. In our procedure, we assumed that the cells that do not die in response to a particular concentration are indeed resistant to that concentration and decided to develop these cells as the resistant cells. We were seeking for the highest concentration of sunitinib that would kill most of the cells, but not all. In this regard, 10 μM sunitinib killed most of the cells (approximately 98%) within 72 h and the remaining 2% of cells were developed to sunitinib resistance. At this stage, the most common practice is to provide a drug holiday period in which the drug is removed from the culture, and the cells are allowed to recover and are rechallenged again (38, 39). Thus, cells undergo multiple cycles of drug rechallenge and drug holiday period before being developed into resistant cells. In our method, the cells were never without drugs and it took approximately 12 months to develop stable sunitinib-resistant cells. Although they were developed with 10 μM sunitinib, they were eventually resistant to up to 40 μM sunitinib. The experiments were performed with 10 μM sunitinib because, translationally, dose escalation is not a common practice and could cause much toxicity.

Morphologically, all resistant cell lines showed hypertrophy. Hatakeyama et al. (40) reported similar findings for 786-0 cells in which the nuclei of sunitinib-resistant cells were increased threefold when compared to parental cell lines; however, the size of the whole cells was not reported in this study. The relevance of hypertrophy in sunitinib-resistant cells is not clear. It could be an adaptation of the cells to redistribute the drug so that the overall intracellular drug concentration is less thus somehow “diluting” the effect of the drug. However, the flaw in this argument is that it could also work the other way around, for example, more surface area is present to absorb more drug. The intracellular concentration of sunitinib in these cells is worth pursuing. Nuclear atypia, including larger size and multilobulation (or nuclear bleb formation) as seen in SN12K1 cells, is not uncommon in laminopathies and cancers, and it is associated with high-grade cancers (41).

Despite larger nucleus or nuclear atypia, the surprising finding was the lack of functional aspects in resistant cells that are often considered as “aggressive” in cancer biology, as they did not show any significant difference when compared with the parental cells. For example, Burtz et al. (42), in an in vivo experimental model, reported that sunitinib-resistant renal tumours exhibited aggressive behaviour such as sarcomatoid differentiation, extensive vascular and local invasion, and liver and lung metastases. In our study, resistant cells did not show any significant changes in growth or transmigration when compared with parental cells. Our results on growth are in agreement with the report of Sakai et al. (43) who developed ACHN cells that are resistant to 10 μM sunitinib. Taken together, the functional data appear to suggest that resistance is not necessarily a concern in terms of aggressiveness. Perhaps, these are simply non-responsive cells without any added aggression. This proposal warrants further exploration.

RCC is a highly heterogeneous disease. While no significant heterogeneity could be observed on the functional aspects, it was obvious in the molecular signature. IL-6 was the only molecule that was increased in all cells. As IL-6 is a regulator of VEGF, with the consensus on a mechanism of angiogenesis restoration or neovascularisation (22, 39, 44), the expression of VEGF was assessed. Interestingly, only two cell lines (Caki-2 and SN12K1) had a significant increase in VEGF mRNA. Thus, VEGF, or angiogenesis, is not necessarily the sole mediator of resistance. To find alternative mechanisms, we explored molecules that regulate apoptosis because apoptosis has been reported to be a mechanism of cell death in RCC in response to sunitinib treatment (45, 46). The results showed that the equilibrium was shifted in favour of the anti-apoptotic molecule Bcl2. Thus, it is not only angiogenesis but also resistance to apoptosis that appear to play a role. To the best of our knowledge, this is the first experimental evidence for the role of anti-apoptosis mechanisms in sunitinib resistance of RCC.

## Conclusion

The question arises regarding the best way to combat resistance. One way is to combine other antiangiogenic agents with sunitinib. Although clinical trials combining bevacizumab and sunitinib showed anti-tumour activity, toxicity precludes further clinical development (47–49). Bcl2 inhibition has long been tried but has not progressed beyond experimental stage (50). Inhibition of IL-6 could be a promising way to overcome drug resistance. In this regard, a recent study showed the beneficial effect of IL-6 receptor inhibition to overcome sunitinib resistance (28). Combination of sunitinib with the ILR-6 inhibitor tocilizumab to overcome sunitinib warrants further research.

## Conflict of interest

The authors declare no conflicts of interest with respect to research, authorship and/or publication of this article.
